# Essential Oil from the Leaves, Fruits and Twigs of *Schinus terebinthifolius*: Chemical Composition, Antioxidant and Antibacterial Potential

**DOI:** 10.3390/molecules29020469

**Published:** 2024-01-17

**Authors:** Kátia C. Oliveira, Lidaiane M. S. S. Franciscato, Suelen S. Mendes, Francielly M. A. Barizon, Daniela D. Gonçalves, Lidiane N. Barbosa, Maria G. I. Faria, Juliana S. Valle, Rhaira F. A. Casalvara, José E. Gonçalves, Zilda C. Gazim, Suelen P. Ruiz

**Affiliations:** 1 Graduate Program in Biotechnology Applied to Agriculture, Universidade Paranaense (UNIPAR), Umuarama 87502-210, PR, Brazil; katia.oliveira@edu.unipar.br (K.C.O.); lidaiane.franciscato@edu.unipar.br (L.M.S.S.F.); suelen.mendes@edu.unipar.br (S.S.M.); gracielaiecher@prof.unipar.br (M.G.I.F.); jsvalle@prof.unipar.br (J.S.V.); cristianigazim@prof.unipar.br (Z.C.G.); 2 Graduate Program in Animal Science with Emphasis on Bioactive Products, Universidade Paranaense (UNIPAR), Umuarama 87502-210, PR, Brazil; francielly.alm@edu.unipar.br (F.M.A.B.); danieladib@prof.unipar.br (D.D.G.); lidianebarbosa@prof.unipar.br (L.N.B.); 3 Graduate Program in Medicinal Plants and Herbal Medicines in Basic Health Care, Universidade Paranaense (UNIPAR), Umuarama 87502-210, PR, Brazil; 4 Graduate Program in Clean Technologies, Cesumar Institute of Science, Technology and Innovation, Cesumar University (UniCesumar), Maringá 87050-390, PR, Brazil; rhaira.casalvara@gmail.com (R.F.A.C.); jose.goncalves@unicesumar.edu.br (J.E.G.)

**Keywords:** Anacardiaceae, pink pepper, β-pinene, caryophyllene, antimicrobial, food safety

## Abstract

*Schinus terebinthifolius* Raddi, popularly known as “Pink pepper”, is a plant native to Brazil. The objective of this work was to analyze the chemical composition and the antioxidant and antibacterial potential of essential oils (EOs) from the leaves, fruits and twigs of *S*. *terebinthifolius,* aiming for their application in food safety. EOs were obtained by hydrodistillation and the chemical composition was determined by gas chromatography coupled to mass spectrometry. Phenolic compounds were quantified and antioxidant activity was evaluated using three different methods. The antibacterial activity was determined by the broth microdilution method against foodborne bacteria. In the chemical analysis, 22 compounds were identified in the leaves, 13 compounds in the fruits and 37 compounds in the twigs, revealing the presence of the main compounds germacrene D (12.04%, 15.78%, 20,41%), caryophyllene (15.97%, 3.12%, 11.73%), α-pinene (11.6%, 17.16%, 2.99%), β-pinene (5.68%, 43.34%, 5.60%) and γ-gurjunene (16,85%, 3,15%) respectively. EOs showed better antioxidant potential using the β-carotene/linoleic acid method with 40.74, 61.52 and 63.65% oxidation inhibition for leaves, fruits and twigs, respectively. The EO from the leaves showed greater antibacterial potential against *Escherichia coli* and *Staphylococcus aureus* with a minimum inhibitory concentration (MIC) of 0.62 mg mL^−1^, a value lower than the MIC of sodium nitrite (5.00 mg mL^−1^), the antimicrobial standard synthetic. The activities of pink pepper EOs suggest their potential as a biopreservative in foods.

## 1. Introduction

The food industry needs to explore new strategies due to the problems caused by the microbial contamination of food, ranging from spoilage to disease outbreaks. Furthermore, there is interest in reducing the use of synthetic chemical additives in food preservation as they can lead, depending on the concentration, to harmful effects on human health such as allergies and carcinogenicity [1]; the aim is replacement with efficient and safe natural substances [2,3].

According to the World Health Organization (WHO), foodborne illnesses are a major global public health concern. There are more than 200 types of foodborne illnesses and it is estimated that every year, one in ten people fall ill as a result of these diseases. Furthermore, foodborne illnesses can be fatal, especially in children under 5 years of age, causing 420,000 deaths per year [4]. Among the main microorganisms that cause foodborne illnesses, the species of bacteria *Pseudomonas aeruginosa*, *Salmonella* Typhi, *Bacillus cereus*, *Escherichia coli* and *Staphylococcus aureus* are reported [5,6,7].

In addition to microbial deterioration of food, chemical changes due to the oxidation of lipid components of food involve the reaction of unsaturated fatty acids with oxygen, leading to the formation of reactive species such as free radicals, peroxides, hydroperoxides and various other compounds, which cause changes in the characteristic sensorial and nutritional value, in addition to the emergence of potentially toxic substances [8]. Furthermore, reactive oxygen species can attack biological macromolecules in vivo in human or animal organisms, which will cause damage to proteins, lipids and DNA, promoting cellular aging and diseases [9]. Studies have reported that these reactions are linked to diseases such as cancer, atherosclerosis, diabetes, arthritis, AIDS, cardiovascular diseases and diseases of aging [10].

The main synthetic antioxidants used in the food industry are butylated hydroxyanisole (BHA), butylated hydroxytoluene (BHT) and tert-butyl hydroquinone (TBHQ) [11], but when in inadequate concentrations, they can cause effects toxic effects on the body such as carcinogenicity, cytotoxicity, induction of oxidative stress and endocrine disruption effects [12].

*Schinus terebinthifolius* Raddi is a plant popularly known as red pepper, Brazilian pepper and pink pepper [13] belonging to the Anacardiaceae family, native to Brazil and widely found in the northwest region of Paraná [14,15,16]. The medicinal and popular uses of this plant (leaves and fruits) have been reported in ethnopharmacological studies as anti-inflammatory, healing and the treatment of respiratory diseases [17,18,19,20]. It is sold and used in dehydrated form, being well accepted in cooking as a gourmet spice [21,22]. The EO extracted from pink pepper fruits has antioxidant, antimicrobial and insecticidal properties [23,24]. These properties may be related to the presence of biologically active compounds in the composition such as germacrene-D, sabinene and pinene [24,25].

Considering the interest in the potential of EOs, studies on pink pepper were carried out to evaluate chemical composition [26,27], antibacterial activity [23,28] and antioxidant activity [29]; however, they did not evaluate EOs from different parts (leaves, fruits and twigs) of the same studied plant in order to understand the distributions of the compounds and their activities. Therefore, the objective of this work was to identify the chemical composition and the antioxidant and antibacterial potential of the EOs from the leaves, fruits and twigs of *S. terebinthifolius*.

## 2. Results

### 2.1. Yield and Chemical Composition of Essential Oils

The yield of essential oils from pink pepper leaves and twigs was 0.63 ± 0.01% (m/m) and 0.12 ± 0.02% (m/m), respectively, while the fruits demonstrated a greater yield of 7.60 ± 0.03% (m/m) (*p* < 0.05).

The results of the chemical identification of the essential oil from the fruits of *S. terebinthifolius* are shown in Table 1. A total of 22 compounds were identified in the leaves, 13 compounds in the fruits and 37 compounds in the twigs. Of the 37 compounds identified in the twigs, 19 were exclusive to this part of the plant. The fruits and leaves presented two and four exclusive compounds, respectively. The predominant class in the leaves and twigs was hydrocarbon sesquiterpenes (69.52% and 64.96%, respectively) and in the fruits, hydrocarbon monoterpenes predominated (63.27%). The major compounds were β-Pinene in the fruits (43.34%), while Germacrene D was the major compound in the twigs (20.41%), and in the leaves, caryophyllene (15.97%) and γ-Gurjunene (16.85%).

**Figure 1:**
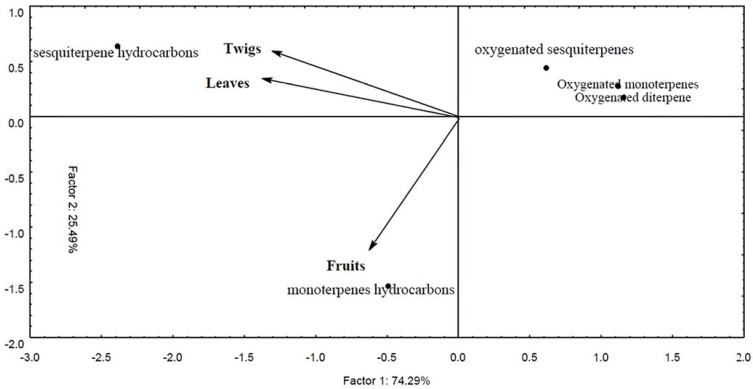
Biplot with representation of the projection of classes of compounds of essential oils from the leaves, fruits and twigs of *Schinus terebinthifolius* obtained by gas chromatography coupled to mass spectrometry (GC/MS).

The results of the chromatographic analyses were subjected to multivariate analysis using the Principal Component Analysis (PCA) technique. According to Figure 1, two classes stood out as the main ones, the hydrocarbon sesquiterpenes in the leaves and twigs and the monoterpenes in the fruits, corroborating the data shown in Table 1.

### 2.2. Antioxidant Activity

The results of antioxidant activity using the DPPH, FRAP and total phenol content methods are presented in Table 2. The essential oil from the leaves showed the highest antioxidant activity by the DPPH method (IC_50_ 5.368 ± 0.132 mg mL^−^^1^).

Regarding the FRAP method, there was no significant difference (*p* < 0.05) between the essential oil extracted from the leaves (0.434 ± 0.005 μM/mL), fruits (0.438 ± 0.002 μM mL^−^^1^) and twigs (0.437 ± 0.004 μM mL^−^^1^). When compared to the positive control Trolox (9.175 ± 0.01 μM mL^−^^1^), they showed reduced antioxidant activity.

The total phenol content in the essential oil varied in relation to the different parts of the plant, being higher in the fruits (24.79 ± 0.37 µg EAG mg^−^^1^).

The results of inhibition (%) of the oxidation of *S. terebinthifolius* EOs by the β-carotene/linoleic acid co-oxidation system are shown in Table 3. The increase in concentration contributed to an increase in the antioxidant activity of the essential oils of fruits (61.52%) and leaves (63.65%) in the concentration of 1.0 mg mL^−^^1^. Compared to the Trolox standard (76.32%), the antioxidant activity of these samples represents 19% and 17% lower activity, respectively.

### 2.3. Antibacterial Activity

The results of the antibacterial activity for the minimum inhibitory concentration (MIC) and minimum bactericidal concentration (MBC) of the essential oils from the fruits, leaves and twigs of *S. terebinthifolius* and the positive control sodium nitrite are shown in Table 4. The MIC values for the essential oil ranged from 5.00 to 10.00 mg mL^−^^1^ for the fruits, 0.62 to 2.50 mg mL^−^^1^ for the leaves and 2.50 to 20.00 mg mL^−^^1^ for the twigs of *S. terebinthifolius*. For the positive control sodium nitrite, the result was 5.00 mg mL^−^^1^ for all species evaluated. For the fruit’s essential oil, the lowest MIC value was against *P. aeruginosa* (5.00 mg mL^−^^1^), being equal (*p* > 0.05) to the MIC obtained for the sodium nitrite control. For the essential oil from the leaves, the lowest MIC values were against *E. coli* (0.62 mg mL^−^^1^) and *B. cereus* (0.62 mg mL^−^^1^), this value being eight times lower than the MIC of the positive control sodium nitrite. For the essential oil from the twigs, the lowest MIC value was for *S. aureus* (2.50 mg mL^−^^1^), two times lower than that of the sodium nitrite control.

The results of the antibacterial activity for the minimum bactericidal concentration (MBC) of the essential oil from the fruits, leaves and twigs of *S. terebinthifolius* and the positive control sodium nitrite are shown in Table 4. The average MBC values ranged from 10.00 to >20.00 mg mL^−^^1^ for fruits, 2.50 to >20.00 mg mL^−^^1^ for leaves and 10.00 to >20.00 mg mL^−^^1^ for the essential oil from the twigs. For the control, sodium nitrite’s MBC was >20.00 mg mL^−^^1^ for all species. For the essential oil of the fruits, the lowest MBC values were against *B. cereus* and *P. aeruginosa* with an average value of 10.00 mg mL^−^^1^, showing greater bactericidal potential than the sodium nitrite control. In relation to the essential oil of the leaves, the lowest MBC was against *P. aeruginosa* (2.50 mg mL^−^^1^), demonstrating greater potential than the sodium nitrite control. For the essential oil from the twigs, the lowest value was for *S. aureus* (10.00 mg mL^−^^1^), also demonstrating greater potential than the sodium nitrite control.

## 3. Discussion

The yield of EOs may vary depending on plant characteristics, collected parts and genetic conditions [30]. In the present study, the yields of EOs extracted from different structures of *S*. *terebinthifolius* varied among themselves and these results are in line with those obtained by Bortolucci et al. [24], who found yields of 7.25 ± 0.61% (m/m) and 0.57 ± 0.10% (m/m) in the fruits and leaves of *S*. *terebinthifolius*, respectively. No reports were found in the literature on the yield of essential oil from *S*. *terebinthifolius* twigs. According to the European Pharmacopoeia, the minimum extraction yield of EOs for development and application in products is 2 mL kg^−1^ [31]. The yield of EOs in the present study was 75.0 mL kg^−1^ from the fruits, 6.3 mL kg^−1^ from the leaves and 1.2 mL kg^−1^ from the twigs, indicating that the essential oils from the fruits and leaves are found within the recommendations stipulated by the European Pharmacopoeia [32], allowing them to be recommended for application in products.

The chemical composition of the essential oil can be directly affected by factors such as the geographic origin of the crop, cultivation method, harvest time, phenological stage, climate, season, part of the plant, whether the plant was dehydrated or fresh, among others [30]. Mohamed et al. [25], in research carried out in Egypt, identified the main compounds in the essential oil from the twigs of *S*. *terebinthifolius* as terpinen-4-ol (18.25%), cis-β-terpineol (15.60%), γ-terpinene (12.46%), sabinene (9.83%), α-terpinene (8.56%) and 4-thujanol (6.71%).

Bortolucci et al. [24] also investigated the chemical composition of the essential oils from the leaves and fresh fruits of *S*. *terebinthifolius* and reported sesquiterpene hydrocarbons as the majority class, the main ones being bicyclogermacrene (27.57%), β-phellandrene (7.30%), germacrene D (7.16%) for leaves and β-pinene (30.32%), germacrene D (14.23%), bicyclogermacrene (5.97%) and α-pinene (3.58%) for the fruits. Bendaoud et al. [33], in a culture established in southern Tunisia, found the main compounds from the fruits of *S. terebinthifolius* to be α-phellandrene (34.38%), γ-cadinene (18.04%), β-phellandrene (10.61%), p-chymene (7.34%), α-pinene (6.49%) and β-pinene (3.09%).

Due to the variety of substances present in the EO of *S*. *terebinthifolius* leaves, the application of a single methodology does not present accurate results on antioxidant activity. In addition, some criteria must be taken into account, such as the type of sample, extraction technique, and chemical components present, as well as methodological parameters such as time, temperature, oxidation time, and mechanism of action [34]. Therefore, in this work, different methodologies (DPPH, FRAP and β-carotene/linoleic acid co-oxidation) were applied to evaluate the antioxidant activity of *S*. *terebinthifolius* essential oils.

The DPPH method is more suitable for extracts or polar substances extracted from aromatic plants, which generally have greater antioxidant activity than essential oils. This fact can be attributed to the presence of phenolic compounds in polar extracts. The non-polar nature of essential oils may explain the low efficiency in stabilizing the hydrophilic DPPH radical [35], which may also justify the low antioxidant activity present in the study. Dannenberg et al. [36] reported an average IC_50_ value of 0.5 ± 0.0008 mg mL^−1^ for essential oil from the green fruit and 0.02 ± 0.0012 mg mL^−1^ for the essential oil from the ripe fruit of *S*. *terebinthifolius*, demonstrating greater antioxidant potential for ripe fruits in this study. Carneiro et al. [37] reported average IC_50_ values of 0.0035 ± 0.0002 mg mL^−1^ and 0.0441 ± 0.0002 mg mL^−1^ for leaves and ripe fruit from *S. terebinthifolius*, respectively. The difference in antioxidant activity observed in the current study may be attributed to differences in the compounds present in the EOs when compared to the referenced studies. When comparing the antioxidant activity of the essential oils from the leaves, fruits and twigs of *S*. *terebinthifolius* in the present study with the activity of the synthetic additive BHT (IC_50_ of 19.8 ± 0.5 mg mL^−1^) reported by Sokmen et al. [38], it can be considered that essential oils presented superior activities. BHT is a synthetic antioxidant compound that can have toxicological effects such as cancer and liver damage [39], and the search for new substances with a view to its industrial replacement is of interest. The FRAP assay is a quick and easy method to perform. Its reaction is reproducible and related to the molar concentrations of the antioxidant in it [40]. However, in the present study, essential oils showed reduced activities compared to the control.

The β-carotene/linoleic acid methodology is more effective for evaluating lipophilic antioxidant activity, such as that of essential oils [41]. According to Hassimotto, Genovese and Lajolo [42], the antioxidant capacity using this method can be classified as high when it results in greater than 70% inhibition, intermediate between 40 and 70% and low when it is less than 40%. Therefore, the essential oils from the leaves and fruits in the present study can be classified as having intermediate activity.

The antioxidant potential found may be related to the chemical compounds identified in the essential oils of the fruits, leaves and twigs of *S*. *terebinthifolius*. However, it can hardly be attributed to their components in isolation, as their chemical compositions present molecules with different functional groups. The synergistic interaction of all components of the essential oils can also justify the antioxidant activity presented [43].

Regarding antibacterial activity, Dannenberg et al. [36] reported the essential oil activity of green and ripe fruits of *S*. *terebinthifolius* Raddi, which showed MICs of 6.79 mg mL^−1^ and 1.74 mg mL^−1^ against *S*. *aureus* (ATCC 6538), respectively, and an MIC of 0.85 mL^−1^ for *B*. *cereus* (ATCC 11778), showing that the oil from the ripe fruit has greater action against bacteria. In a study also carried out by Dannenberg et al. [26], they determined the chemical composition of the essential oil from the ripe fruits of *S*. *terebinthifolius* and evaluated the antibacterial activity against *S*. *aureus* (ATCC 6538) and *Listeria monocytogenes* (ATCC 7644), which they identified as the major compounds β-myrcene (41%), β-cuvebene (12%) and limonene (9%), and they reported MIC values of 0.68 and 1.32 mg mL^−1^ for *S*. *aureus* and *L*. *monocytogenes*, respectively. When compared with the literature, the results of the present study showed higher MIC values, suggesting that these seasonal variations in the composition of essential oils and their allelopathic activity may be related to the origin, season, and cultivation conditions of the plant and whether the fruit is green or ripe.

Gram-positive bacteria generally present greater susceptibility to essential oils than Gram-negative bacteria due to the complexity of the constitution of the cell wall, such as the presence of lipopolysaccharides, which makes it difficult for the essential oil to penetrate the cell [44]. In the present study, essential oils from different parts of *S*. *terebinthifolius* showed activity for both Gram-positive and Gram-negative bacteria. The activity of essential oils also cannot be confirmed based on a single mode of action, due to the various compounds present in the plant material [45]. Their action can be attributed to the degradation of the cell wall, penetration of the bacterial cell membrane and inhibition of its functional properties, coagulation and inhibition of cytoplasmic enzymes and depletion of the proton motive force [45,46].

The essential oils extracted from leaves, fruits and twigs of *S*. *terebinthifolius* showed the presence of different chemical components, which explains the different effects in inhibiting bacterial growth. The antibacterial activity can also be attributed to the possible synergistic effect between the components of essential oils. When evaluated separately by Souza et al. [46], the positive enantiomer of α-pinene showed antibacterial activity against strains of *S*. *aureus* (ATCC 25923) and *E*. *coli* (ATCC 25922), suggesting that (+) α-pinene may be a compound used as an antimicrobial in the future [47].

The β-caryophyllene isomer, one of the major compounds present in this study, is one of the main components of essential oils extracted from spices and food plants [48,49]. This plant compound has been approved by the Food and Drug Administration (FDA) and the European Food Safety Authority (EFSA) and is used as a flavor enhancer in foods [50].

It is important to highlight that the essential oils from different parts of the plant had greater antibacterial potential than the preservative sodium nitrite. Sodium and potassium nitrite are widely used as food additives with the purpose of preserving, intensifying or modifying the sensory properties of foods [51]. The ingestion of nitrites can cause hemolymphatic problems, since the nitrite toxicity mechanism acts on the oxygen transport process, acting on hemoglobin and producing methemoglobin, which prevents the transport of oxygen from the alveoli to the tissues, which may lead the individual to death [52,53]. Nitrite also reacts with amines in the human body and can form nitrosamines, which are potentially carcinogenic [54,55]. Therefore, the search for natural substances with preservative potential is a promising way to minimize the toxic effects caused by food additives, showing that the use of essential oils is promising in the food industry. The results obtained in this work prove the potential of *S*. *terebinthifolius* essential oil, as a source of active compounds regarding antioxidant and antimicrobial properties, to be possibly used as an alternative to traditional antimicrobials, and they open the field for new research, as well as new tests for applications as a biopreservative in foods.

## 4. Materials and Methods

### 4.1. Plant Material

The leaves, ripe fruits and twigs of *S*. *terebinthifolius* were collected in Umuarama, Brazil, at the geographic coordinates 23°66′0.27″ S, 53°301′45″ W in February 2020. An exsicata was deposited in the Herbarium of the Horto Medicinal da Universidade Paranaense under number 364. This species was registered in the National Genetic Heritage and Associated Traditional Knowledge Management System (SisGen) under registration number ABF50ED.

### 4.2. Extraction of Essential Oils from the Leaves, Fruits and Twigs of Schinus terebinthifolius

Two hundred and fifty grams of fresh plant material were used for 2.5 L of distilled water. The different parts of the plant (leaves, fruits and twigs) were separately crushed in an industrial blender and subjected to the hydrodistillation process in a modified Clevenger apparatus for 2 h [56]. After this period, the essential oils were removed from the extractor with the aid of a Pasteur pipette and filtered with anhydrous sodium sulfate (Na_2_SO_4_). They were then placed in amber bottles, weighed and kept refrigerated at 4 °C. The essential oil yield was obtained according to Equation (1):

Essential oil content (%) = Essential oil mass (g)/Plant mass (g) × 100 (1)

The concentrations of EOs for the assays were calculated based on fresh materials.

### 4.3. Analysis of the Chemical Composition of Essential Oils

Chemical identification was carried out on a gas chromatograph (Agilent 7890 B, Agilent Technologies, Santa Clara, CA, EUA) coupled to a mass spectrometer (Agilent 5977A, Agilent Technologies, Santa Clara, CA, EUA), equipped with an Agilent HP-5MS UI (Agilent Technologies, Santa Clara, CA, EUA) fused silica capillary column (30 m × 0.250 mm × 0.25 μm). The analysis conditions were as follows: injector temperature of 260 °C, injection volume of 2 µL, injection ratio in split mode 1:30, initial column temperature of 60 °C remaining for 2 min, with a heating ramp of 2 °C/min up to 180 °C remaining for 4 min, a ramp of 10 °C/min to 260 °C for 10 min and finally a ramp of 40 °C/min to 300 °C for 1 min. The transfer line was maintained at 260 °C and the ionization source and quadrupole at 230 °C and 150 °C, respectively. He gas was used as a carrier gas with a flow rate of 1 mL/min. The detection system was EM in Scan mode, in the mass/charge ratio range (*m*/*z*) of 40–550, with a Solvent Delay of 3 min. The oil samples were diluted in a ratio of 1:10 with dichloromethane. The compounds present in the essential oils were identified by comparing their mass spectra with the mass spectra of the NIST 11.0 library and based on the comparison of their retention indices (RI) obtained using a series homologous to the n-alkane standard (C7–C40) [57].

### 4.4. Analysis of the Main Components of Essential Oils

A multivariate exploratory analysis was carried out, determining the principal component analysis (PCA), which allowed the joint assessment of the chemical class of all compounds present in the essential oils of leaves, fruits and twigs. The result of the analysis was presented in graphic form (Biplot), helping to characterize the groups of variables analyzed [58]. For each sample of essential oil obtained from leaves, fruits and twigs, an exploratory analysis of chemical classes was carried out as well as the relative area (%). These data were transformed into orthogonal latent variables called main components, which are linear combinations of the original variables created with the eigenvalues of the data’s covariance matrix [59]. Kaiser’s criterion was used to choose the main components, with an eigenvalue preserving relevant information when it is greater than unity [60,61]. This analysis was carried out by referring to the chemical classes of the compounds using the Statistica 7 program [62].

### 4.5. Antioxidant Activity

#### 4.5.1. Determination of Total Phenol Content (FT)

The determination of the total phenol content present in the essential oils of the leaves, fruits and twigs of *S*. *terebinthifolius* was carried out using spectroscopy in the visible region using the Folin–Ciocalteu method according to Swain and Hills [63] with modifications [64]. The samples were diluted in methanol at a concentration of 1.0 mg mL^−1^. The reagent solution was composed of 155 μL of the Folin–Ciocalteu solution, 125 μL of sodium carbonate solution followed by 20 μL of the diluted sample (1 mg mL^−1^) in each well of the microplate. The mixture was left to rest in the absence of light for 60 min and the reading was performed on a SpectraMax Plus384 Microplate Reader at 760 nm, in triplicate. The calibration curve was obtained using seven dilutions of gallic acid (0–100 µg mL^−1^). The calibration curve equation obtained by linear regression occurred according to Equation (2):

A = 0.0196 C − 0.031    (R^2^ = 0.9997) (2)

where A represents the measured absorbance, C represents the concentration of gallic acid equivalents and R^2^ represents the coefficient of determination for the multiple regression. Results were expressed as µg gallic acid equivalent (GAE) mg^−1^ of sample.

#### 4.5.2. Free Radical-Scavenging Method 2,2 Diphenyl-1-picrylhydrazyl (DPPH)

To determine the free radical-scavenging capacity of DPPH, the methodology described by Rufino et al. was used [65]. An aliquot of 10 µL of samples of essential oils from the leaves, fruits and twigs of *S*. *terebinthifolius* at concentrations of 1.00, 0.75, 0.50 and 0.25 mg mL^−1^ were added to 290 µL of methanolic DPPH solution (60 µM). For the negative control, 10 µL of methanol was used in a DPPH solution (60 µM). The mixture was kept in the dark at room temperature for 30 min. The reduction in absorbance was measured at 515 nm on a SpectraMax Plus384 Microplate Reader. The total antioxidant capacity of the extracts was calculated using a standard quercetin solution (60 µM) as a 100% reference. From the correlation between absorbance and the concentration of the antioxidant sample, the concentration necessary to reduce 50% of free radicals was determined (IC_50_).

#### 4.5.3. β-Carotene/Linoleic Acid Co-Oxidation System

The antioxidant capacity of essential oils from the leaves, fruits and twigs *of S. terebinthifolius* was evaluated according to Rufino et al. [66]. The reaction was monitored by spectrophotometry, measuring the loss of β-carotene color. In a beaker, 20 μL of linoleic acid, 265 μL of Tween 40, 25 μL of the β-carotene solution (20 mg mL^−1^) and 0.5 mL of chloroform were added. The solvent was removed using a dryer and then the mixture was dissolved in 20 mL of hydrogen peroxide. The antioxidant activity of the samples was determined by mixing 280 μL of emulsion with 20 μL of samples at different concentrations (1.00, 0.75, 0.50 and 0.25 mg mL^−1^). Samples were kept for 120 min and readings were measured at an absorbance of 470 nm. A Trolox solution was used as a control. The results were expressed as a percentage of oxidation inhibition, following Equation (3), and the reduction in absorbance of the antioxidant system was considered as 100% oxidation. From the absorbance value following Equation (4), the percentage of oxidation correlated with the absorbance of the sample was calculated, decreasing with the absorbance of the system, and the percentage of oxidation of each sample was subtracted from 100 (Equation (5)) to obtain the percentage of oxidation inhibition (%).

Absorbance reduction = Abs_initial_ − Abs_final_ (3)

% Oxidation = [(Reduction Abs) sample × 100]/(Reduction Abs) system (4)

% Protection = 100 − (%Oxidation) (5)

#### 4.5.4. Ferrous Reduction Method (FRAP)

The methodology used was described by Benzie [67] and modified by Rufino et al. [68]. For the FRAP (Ferric-Reducing Antioxidant Power) method, 25 mL of acetate buffer (0.3 M) and 2.5 mL of an aqueous solution of 2,4,6-Tris (2-pyridyl)-s-triazine (TPTZ—10 mM) and 2.5 mL of an aqueous solution of ferric chloride (20 mM) were used. For the antioxidant activity reaction, a microplate with 96 wells was used where 10 μL of essential oils from the leaves, fruits and twigs of *S*. *terebinthifolius* were added at concentrations of 1.00, 0.75, 0.50 and 0.25 mg mL^−1^ along with 290 μL of FRAP reagent. The plate was placed in the Spectra Max Plus384 equipment, homogenized vigorously by the equipment and maintained at 37 °C for 30 min. The change in absorbance was read at 595 nm. The percentage of antioxidant activity was calculated relative to a standard curve of ferrous sulfate (1000 μM). Antioxidant activity was expressed as μM ferrous sulfate/mg of sample.

### 4.6. Antibacterial Activity

#### 4.6.1. Microorganisms and Inoculum Preparation

The antibacterial activity of the essential oil was tested against five bacterial strains: *Bacillus cereus* (ATCC 14579), *Escherichia coli* (ATCC 43893), *Salmonella enterica* subsp. *enterica* Typhi (ATCC 19214), *Staphylococcus aureus* (ATCC 25923) and *Pseudomonas aeruginosa* (ATCC 27853). Bacterial cell mass dilution from 24 h culture was prepared for the assays. The final bacterial cell concentration was adjusted in a spectrophotometer at 625 nm using 0.85% (*m*/*v*) sterile saline and adjusted to the 0.5 McFarland Scale (1.5 × 10^8^ CFU mL^−1^). Then, the suspension was diluted to 1:10 in Muller Hinton Broth culture medium, obtaining 1.5 × 10^7^ CFU mL^−1^ as inoculum, which was used to determine the minimum inhibitory concentration (MIC).

#### 4.6.2. Antibacterial Activity by Broth Microdilution Method

The MIC of the essential oils was determined by serial microdilution in 96-well microplates according to the broth microdilution method [69], as modified for natural products. The tested concentrations of essential oils ranged from 20.00 to 0.039 mg mL^−1^, which were dissolved in distilled water and 2% Tween 80 and subsequently evaluated in a total volume of 100 μL of the solution (culture medium and samples). Positive sodium nitrite control (50 to 1.25 mg mL^−1^) dissolved in sterile distilled water was used to assess antibacterial activity. After serial dilution, 50 µL of the inoculum 1.5 × 10^5^ CFU mL^−1^ prepared in saline solution, as described in the previous item, was added to each well (1.5 × 10^5^ CFU mL^−1^) and subjected to incubation at 35 °C for 24 h. Reading was performed with the addition of 20 μL of 1.0% 2,3,5-triphenyltetrazolium chloride (Êxodo Científica®, Sumaré, São Paulo) developer in each well followed by incubation of microplates at 37 °C for 20 min. The MIC was defined as the lowest concentration that resulted in visual growth inhibition, according to the developer. The minimum bactericidal concentration (MBC) was determined by subculturing 10 µL from each well on Muller Hinton agar plates and incubating at 35 °C for 24 h.

### 4.7. Statistical Analysis

All analyses were performed in triplicate. Results were expressed as arithmetical mean and standard deviation. Data were submitted to one-way analysis of variance (ANOVA) followed by Tukey’s test at a 5% level. Statistical analyses were carried out using SISVAR.

## 5. Conclusions

The EO yield of fruits was higher than that of twigs and leaves. The main compounds were germacrene D in leaves (12.04%), fruits (15.78%) and twigs (20.41%); caryophyllene in leaves (15.97%) and twigs (11.73%); α-pinene in leaves (11.6%) and fruits (17.16%); β-pinene (43.34%) in fruits; and γ-gurjunene (16.85%) in the leaves. EO showed potential antioxidant activity using the β-carotene method but reduced activity for the DPPH and FRAP methods and low total phenol content. The antibacterial activity was evident for EO from the leaves, which presented an MIC eight times lower than that of sodium nitrite. This research contributed to the study of identifying the chemical constituents of plant parts, as well as their relationship with biological activities. However, further studies are needed to assess their action in food matrices. In this way, this research opens the field for new research, aiming at the development of products for future applications in food as an alternative for conservation. 

## Figures and Tables

**Table 1:** Chemical identification by gas chromatography coupled to mass spectrometry (GC–MS) of essential oils extracted from the leaves, fruits and twigs of *Schinus terebinthifolius*.

Peak	RT ^1^	Compound	RI ^2^	RI	RA % ^3^		
					Leaves	Fruits	Twigs
1	5.833	α-pinene	934	932	11.6	17.16	2.99
2	7.13	β-pinene	976	974	5.68	43.34	5.60
3	7.13	α-phellandrene	1003	1002	0.61	0.85	0.44
4	8.523	3-carene	1009	1008	0.79	-	0.54
5	8.803	α-terpinene	1014	1014	-	-	0.17
6	9.144	p-cymene	1019	1020	-	-	0.43
7	9.32	D-limonene	1018	1024	-	1.92	1.50
8	9.321	β-phellandrene	1023	1025	0.43	-	0.66
9	9.324	eucalyptol	1025	1026	1.49	-	-
10	10.274	β-cis-ocimene	1039	1032	-	-	0.26
11	10.717	γ-terpinene	1048	1054	-	-	0.40
12	17.051	terpinen-4-ol	1178	1174	-	-	1.18
13	17.882	α-terpineol	1185	1186	-	-	0.42
14	26.657	δ-eIemene	1330	1335	0.71	-	-
15	27.442	α-cubebene	1343	1345	-	-	1.31
16	28.963	α-copaene	1373	1374	5.25	-	5.73
17	29.88	β-cubebene	1383	1387	1.55	-	1.94
18	30.03	β-elemene	1390	1389	4.57	-	1.44
19	31.549	α-gurjunene	1409	1409	2.15	2.12	0.82
20	31.549	caryophyllene	1418	1417	15.97	3.12	11.73
21	32.621	(-)-aristolene	1427	1428	-	-	0.40
22	33.155	α-himachalene	1446	1449	-	-	1.42
23	33.493	α-humulene	1452	1452	1.91	-	1.99
24	34.913	E-β-farnesene	1454	1454	-	-	1.31
25	34.212	allo-aromadendrene	1459	1458	2.25	-	1.49
26	34.928	γ-gurjunene	1475	1475	16.85	3.15	-
27	35.241	γ-muurolene	1476	1478	-	-	1.27
28	35.45	germacrene D	1481	1484	12.04	15.78	20.41
29	36.435	valencene	1495	1496	-	-	6.38
30	36.569	α-selinene	1497	1498	1.33	-	-
31	37.148	α-muurolene	1499	1500	-	-	0.63
32	37.262	δ-amorphene	1509	1511	4.94	3.21	0.77
33	37.811	δ-cadinene	1503	1522	-	-	5.59
34	38.212	cadina-1,4-diene	1510	1524	-	-	0.33
35	39.29	elemol	1530	1548	-	2.7	-
36	40.628	spathulenol	1573	1577	3.75	1.21	5.47
37	40.92	caryophyllene oxide	1578	1582	1.99	0.77	4.83
38	41.041	globulol	1589	1590	1.11	-	-
39	42.167	viridiflorol	1590	1592	-	-	0.61
40	43.577	epicubenol	1603	1617	-	-	0.66
41	44.316	τ-cadinol	1618	1625	1.02	-	2.17
42	44.632	torreyol	1624	1632	-	-	0.39
43	45.046	τ-muurulol	1638	1640	-	-	1.08
44	69.658	mandenol	2151	2159	-	2.02	-
		**Total Identified**			**97.99**	**97.35**	**94.76**
		monoterpenes hydrocarbons			19.11	63.27	12.99
		oxygenated monoterpenes			1.49	-	1.6
		sesquiterpenes hydrocarbons			69.52	30.08	64.96
		oxygenated sesquiterpenes			7.87	1.98	15.21
		diterpene oxygenated			-	2.02	-

^1^ RT = Retention time (min); ^2^ RI = retention index. The compounds were identified by comparing their mass spectra with the mass spectra of the NIST 11.0 library and based on the comparison of their retention indices (RIs) obtained using a series homologous to the n-alkane standard (C7–C40). ^3^ RA = Relative area (%) = Percentage of the area that the compound occupies in the chromatogram; (-) = compound absent.

**Table 2:** Antioxidant activity by the free radical-scavenging method 2.2 diphenyl-1-picrylhydrazyl (DPPH), the iron-reducing power (FRAP) and determination of the phenol content (FT) of the essential oils of the leaves, fruits and twigs of *Schinus terebinthifolius*.

Samples	DPPH	FRAP	Total Phenolics
	IC_50_ (mg mL^−1^)	(µM Ferrous Sulphate mg^−1^)	(µg AGE mg^−1^)
Leaves	5.368 ± 0.132 ^b^	0.434 ± 0.005 ^b^	23.66 ± 1.60 ^b^
Fruits	14.760 ± 0.108 ^d^	0.438 ± 0.002 ^b^	24.79 ± 0.37 ^a^
Twigs	12.690 ± 0.483 ^c^	0.437 ± 0.004 ^b^	19.17 ± 0.72 ^c^
Quercetin	0.01 ± 0.01 ^a^	-	-
Trolox	-	9.175 ± 0.01 ^a^	-

Values are the mean ± standard deviation of the experiment performed in triplicate. The statistical analysis used was analysis of variance (ANOVA), and the differences between the means were determined by the Tukey test (*p* ≤ 0.05). Values in the same column with different letters are significantly different (*p* ≤ 0.05). IC_50_: oil concentration necessary to inhibit 50% of the DPPH radical (2,2 diphenyl-1-picrylhydrazyl); FRAP: ferrous-reducing antioxidant power; AGE: gallic acid equivalents.

**Table 3:** Percentage of inhibition (%) of oxidation by the β-carotene/linoleic acid co-oxidation system of essential oils from the twigs, leaves and fruits of *Schinus terebinthifolius*.

Samples	Concentrations (mg mL^−1^)			
	1	0.75	0.5	0.25
Leave	63.65 ± 1.38 ^dC^	55.23 ± 2.25 ^cB^	50.63 ± 2.75 ^bB^	44.93 ± 2.85 ^aA^
Fruits	61.52 ± 1.13 ^dB^	54.60 ± 1.27 ^cA^	49.69 ± 2.45 ^aA^	54.09 ± 2.94 ^bC^
Twigs	40.75 ± 2.10 ^aA^	58.76 ± 0.71 ^dC^	51.62 ± 2.53 ^cC^	47.95 ± 1.24 ^bB^

Values are the mean ± standard deviation of the experiment performed in triplicate. Values in the same column with different capital letters are significantly different (*p* ≤ 0.05). Values in the same row with different lowercase letters are significantly different (*p* ≤ 0.05). Positive control: Trolox (0.2 mg mL^−1^); inhibition of oxidation of 76.32%.

**Table 4:** Minimum inhibitory concentration (MIC) and minimum bactericidal concentration (MBC) of essential oils from the fruits, leaves and twigs of *Schinus terebinthifolius* and sodium nitrite control.

Bacteria	Leaves	Fruits	Twigs	Sodium Nitrite
	(mg mL^−1^)	(mg mL^−1^)	(mg mL^−1^)	(mg mL^−1^)
	MIC	MIC	MIC	MIC
	MBC	MBC	MBC	MBC
*Staphylococcus aureus*	1.25 ± 0.00 ^b^	10.00 ± 0.01 ^d^	2.50 ± 0.00 ^c^	5.00 ± 0.00 ^c^
	5.00 ± 0.01 ^b^	20.00 ± 0.01 ^d^	10.00 ± 0.00 ^c^	>20.00 ±0.00 ^d^
*Escherichia coli*	0.62 ± 0.00 ^b^	10.00 ± 0.00 ^d^	20.00 ±0.002 ^e^	5.00 ± 0.00 ^c^
	20.00 ± 0.00 ^b^	>20.00 ± 0.00 ^b^	>20.00 ±0.00 ^b^	>20.00 ± 0.00 ^b^
*Bacillus cereus*	0.62 ± 0.00 ^b^	10.00 ± 0.00 ^d^	10.00 ± 0.00 ^d^	5.00 ± 0.00 ^c^
	10.00 ± 0.01 ^b^	10.00 ± 0.00 ^b^	20.00 ± 0.00 ^c^	>20.00 ± 0.00 ^c^
*Salmonella* Typhi	2.50 ± 0.00 ^b^	10.00 ± 0.01 ^d^	>20.00 ± 0.02 ^e^	5.00 ± 0.00 ^c^
	20.00 ± 0.00 ^b^	>20.00 ± 0.00 ^b^	>20.00 ± 0.00 ^b^	>20.00 ± 0.01 ^b^
*Pseudomonas aeruginosa*	2.50 ± 0.00 ^b^	5.00 ± 0.00 ^c^	20.00 ±0.00 ^d^	5.00 ± 0.00 ^c^
	2.50 ± 0.00 ^b^	10.00 ± 0.00 ^c^	20.00 ±0.00 ^d^	>20.00 ± 0.00 ^d^

Values are the mean ± standard deviation of the experiment performed in triplicate. The statistical analysis used was analysis of variance (ANOVA), and the differences between the means were determined by the Tukey test. Values in the same row with different letters are significantly different (*p* ≤ 0.05).

## Data Availability

Data are contained within the article.
